# Carcinosarcoma and sarcomatoid carcinoma of the stomach: Two case reports

**DOI:** 10.1097/MD.0000000000024697

**Published:** 2021-03-26

**Authors:** Youpeng Li, Li Cui, Ying Chen, Furong Wang

**Affiliations:** a Digestive Medical Department, Minqin People's Hospital, Minqin; b Department of Pathology, Lanzhou University Second Hospital, Lanzhou, Gansu Province, China.

**Keywords:** carcinosarcomas, cases report, differential diagnosis, histopathological morphology, sarcomatoid carcinomas, stomach

## Abstract

**Rationale:**

Carcinosarcoma and sarcomatoid carcinoma of the stomach are rare, malignant, and biphasic tumors with high mortality. The differential diagnosis of these 2 diseases remains challenging. In the present study, we present 2 cases of carcinosarcoma and sarcomatoid carcinoma of the stomach.

**Patient concerns:**

A 54-year-old woman was admitted with complaints of epigastric pain for 4 months, but she became serious for 10 days accompanied by melena. A 75-year-old man was admitted with complaints of epigastric pain for 1 month.

**Diagnosis:**

The female had a Borrmann type III irregular ulcerative lesion (5.0 × 4.0 × 1.0 cm) originating from the gastric antrum. The male had Borrmann type I tumor polypoid exophytic (5.0 × 4.0 × 2.0 cm) in the fundus of stomach near the cardia. Both cases were identified as malignant neoplasms by endoscopic biopsy and further confirmed by performing laparoscopic proximal gastrectomy, esophagogastrostomy, and palliative distal subtotal gastrectomy. The postoperative histopathological morphology and immunohistochemistry studies revealed sarcomatoid carcinoma for the female and gastric carcinosarcoma for the male respectively.

**Interventions:**

The female patient subsequently underwent laparoscopy-assisted radical distal gastrectomy for gastric cancer followed by systemic chemotherapy with oxaliplatin plus tegafur. The male patient underwent laparoscopic proximal gastrectomy and esophagogastrostomy were performed.

**Outcomes:**

The female had a mixture of a little poorly-differentiated adenocarcinoma and abundant sarcomatoid spindle cell elements, and is still alive healthy up to date for 2 and a half years after surgery by phone follow-up. The male patient had both adenocarcinoma and fibrosarcoma in a single tumor, and died 1 month after the operation.

**Lessons:**

The present study provides insight into the clinical findings, differential diagnosis, and prognosis of carcinosarcomas and sarcomatoid carcinomas of the stomach. More cases are needed for further studies in the future.

## 1 Introduction

Carcinosarcoma and sarcomatoid carcinomas of the stomach are rare malignant tumors.^[1]^ The carcinosarcoma was composed of carcinoma originating from epithelial tissue and sarcoma originating from the mesenchymal/connective tissue in a single tumor. Sarcomatoid carcinoma is poorly differentiated, and its components can always be observed in the tumor. These tumors can occur in diverse organs, such as the uterus, lungs, ovaries, salivary glands, and pancreas.^[1–5]^ In the upper gastrointestinal system, carcinosarcoma is often found in the esophagus, and is usually composed of square carcinoma and sarcoma. However, gastric carcinosarcoma and sarcomatoid carcinoma have rarely been reported.^[6]^ Meanwhile, carcinosarcoma and sarcomatoid carcinoma are different from other malignant biphasic tumors, such as some collision tumors, primary gastric synovial sarcoma, and gastroblastoma. In the present study, we present 2 rare cases of carcinosarcoma and sarcomatoid carcinoma of the stomach.

## 2 Case presentations

### 2.1 Case 1

A 54-year-old woman was admitted to our hospital on July 1, 2017, with complaints of epigastric pain for 4 months that had been aggravated for 10 days accompanied by melena. She had no family history of cancer. Her hematological data on admission were within the normal range, and the levels of tumor markers were as follows: carcinoembryonic antigen (CEA), 1.42 ng/ml; carbohydrate antigen 19–9 (CA19-9) 8.97 U/ml; alpha-fetoprotein (AFP) 2.15 ng/ml; and carbohydrate antigen 125 (CA125) 12.46 U/ml, all of which were within the normal limits. Chest radiography revealed no abnormalities in the lung fields, soft tissue, or cardiac shadows. Chest computed tomography (CT) showed interstitial changes in the lungs with no metastatic sites. Abdominal magnetic resonance (MR) imaging revealed an irregular thickening wall at the antrum, the descending part, and the bulbar zone of the duodenum. Additionally, there was a nodular contour with obvious intensity in addition to the descending part of the duodenum.

**Figure 1:**
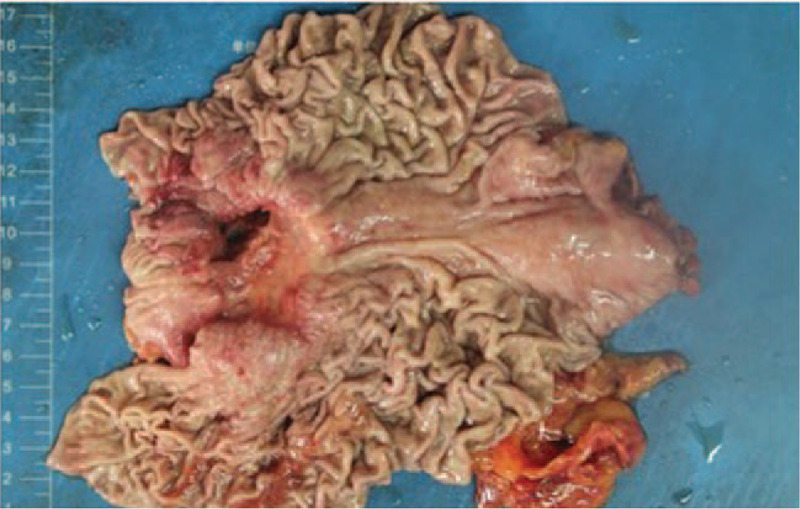
Macroscopic finding. Macroscopic findings revealed a 5.0cm × 4.0cm × 1.0 cm irregular ulcerative lesion (Borrmann type III) originated from the gastric antrum. Surrounding mucosa present irregular convex deforms.

Endoscopic examination revealed a 5.0cm × 4.0cm × 1.0 cm irregular ulcerative lesion (Borrmann type III) originated from the gastric antrum. The surrounding mucosa presented irregular convex deforms (Fig. 1). Preoperative endoscopic biopsy revealed a malignant tumor, mild atrophic gastritis, and mild intestinal metaplasia around the tumor.

The patient subsequently underwent laparoscopy-assisted radical distal gastrectomy for gastric cancer. During operation, an ulcerative lesion with size of 5.0cm × 4.0cm × 1.0 cm was found in the antrum, which invaded the superficial muscular layers of the gastric wall.

**Figure 2:**
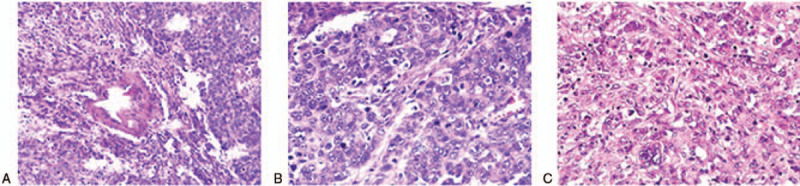
Microscopic findings. Microscopically, the tumor was mainly composed of 2 patterns, a little poorly-differentiated adenocarcinoma and abundant sarcomatoid spindle cell in conventional H&E staining (Magnification: 40 × 10). A: Distinct transitional area was observed between 2 components. B: The adenocarcinoma was arranged broad cords or nests. C: Sarcomatoid spindle cells presented as Reed-sternberg cell features.

Microscopically, the tumor was mainly composed of 2 patterns: a poorly differentiated adenocarcinoma and abundant sarcomatoid spindle cell in conventional hematoxylin and eosin staining. Moreover, a distinct transitional area was observed between the 2 components (Fig. 2A). The adenocarcinoma was arranged in broad cords or nests (Fig. 2B). Furthermore, sarcomatoid spindle cells presented as Reed-Berberg cell features (Fig. 2C), which contained abundant basophilic cytoplasm, different-sized nuclei with prominent giant nucleoli, and vesicular nucleoli. Multinuclear giant cells were observed in the tumor, and many inflammatory cells were also observed in the tumor tissues, including plasma cells, eosinophilia, neutrophils, lymphocytes, and lymphoid follicles. In addition, there were intravascular cancer emboli and massive necrosis in the tumor. The tumor had infiltrated the entire gastric wall. Metastasis was found in 5 of 16 lymph nodes of the gastric omentum, with the main components of sarcomatoid cells. The case had tumor of stage III with TNM clinical classification was T3N1M0.

**Figure 3:**
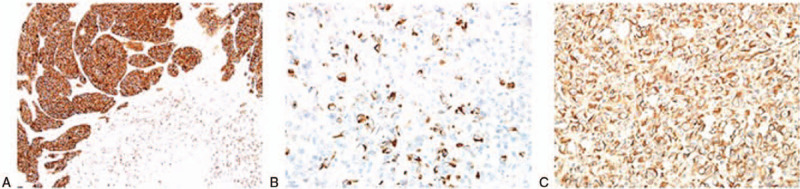
Immunohistochemical findings. Immunohistochemical studies showed that: A: The carcinoma cells were positive for pancytokeratin AE1/AE3 and low molecular weight cytokeratin CK8/18 (Magnification: 20 × 10), B: Negative for mesenchymal marker vimentin (Magnification: 40 × 10) and C: Sarcomatoid carcinoma cells were partially positive for AE1/AE3 and strongly positive for vimentin (Magnification: 40 × 10).

Immunohistochemical studies showed that the carcinoma cells were positive for pancytokeratin AE1/AE3 and low molecular weight cytokeratin CK8/18 (Fig. 3A), and negative for the mesenchymal marker vimentin (Fig. 3B). Sarcomatoid carcinoma cells were partially positive for AE1/AE3 and strongly positive for vimentin (Fig. 3C), whereas all the tumor cells were negative for neuroendocrine markers and other mesenchymal markers, such as desmin, SMA, myglobin, mygenin, S-100, DOG-1, CD34, CD117, CD15, CD30, and CD20. Based on pathological examination, the final diagnosis was sarcomatoid carcinoma.

The patient was discharged on the 15th postoperative day in good condition. Three months after the operation, systemic chemotherapy with oxaliplatin plus tegafur was administered. The patient was still alive and healthy during the telephone follow-up for 3 years.

### 2.2 Case 2

A 75-year-old man was admitted for surgery in our hospital with a 1 month-history of epigastric pain accompanied by melena and fatigue on May 28, 2013. The weight loss was 10 kg over 6 months. He had cholecystitis in 1998 and underwent an open cholecystectomy. In April 2013, the patient did not have a family history of cancer. Most of routine laboratory examinations were found to be in normal range. Some abnormalities were as follows: total protein 52.5 g/L, albumin 26.7 g/L, globulin 25.8 g/L. The patient was positive for occult bleeding. The levels of tumor markers demonstrated normal ranges in carcinoembryonic antigen (CEA, 3.55 ng/ml), alpha-fetoprotein (AFP, 1.69 ng/ml), carbohydrate antigen (CA125) 48.87 U/ml and carbohydrate antigen (CA724, 5.32 U/ml) and the increased carbohydrate antigen (CA19-9, 501.7 U/ml).

Endoscopic examination showed a 5.0cm × 4.0 cm invasive and ulcerative lesion in the posterior wall near the cardia, while its surrounding mucosa was elevated. Gastric cancer (Borrmann I) was also considered. Abdominal computed tomography (CT) revealed an irregular thickening wall with enlarged lymph nodes at the cardia and small cysts in the kidney and liver. The patient was diagnosed with gastric cancer based on the endoscopic biopsy results.

**Figure 4:**
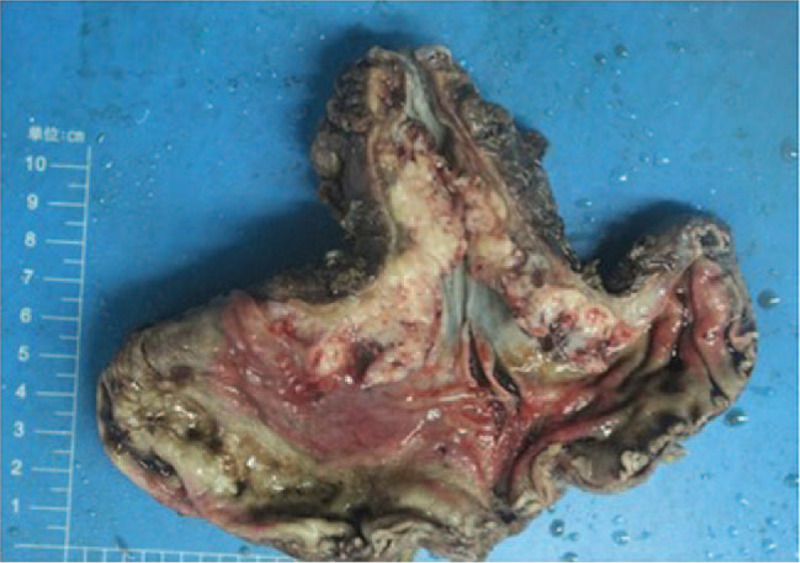
Macroscopic findings. A 5.0cm × 4.0cm × 2.0 cm exophytictumoral mass (Borrmann I) was found, which involved the posterior wall from the cardia to the antrum. Necrosis and haemorrhagia were observed in the tumor.

Laparoscopic proximal gastrectomy and esophagogastrostomy were performed. During the operation, the tumor was observed in the fundus and invaded the stomach wall. The adjacent lymph nodes were swollen. Macroscopically, a 5.0cm × 4.0cm × 2.0 cm exophytictumoral mass (Borrmann I) was found, which involved the posterior wall from the cardia to the antrum. Necrosis and hemorrhage were observed in the tumors (Fig. 4). The tumor had infiltrated all layers of the stomach and was accompanied by obvious necrosis. The tumor did not invade either the esophagus or duodenum. In addition, metastases to other organs were not observed.

**Figure 5:**
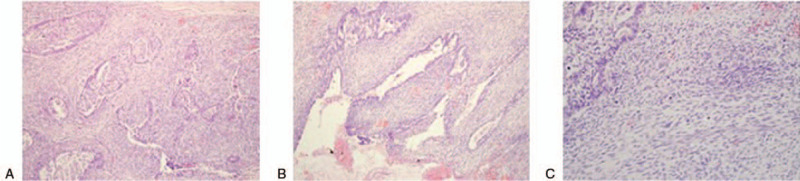
Microscopic findings with H&E staining (Magnification: 40 × 10). A: In the well-differentiated adenocarcinomatous areas characterized by various shapes of tubular structures, these tubular structures were surrounded by fibrosarcoma characterized with infiltrative growth. B: It is looking like phyllodes tumor of the breast. In some areas, the 2 tumor components were separated. C: The sarcoma cells were spindle of same size with frequent mitoses (> 5/HPF) and arrayed as fascicular and interlaced.

The tumor showed mixed well-differentiated adenocarcinoma and fibrosarcoma using conventional hematoxylin and eosin staining. The histology varied greatly. In the well-differentiated adenocarcinomatous areas characterized by tubular structures of various shapes, these tubular structures were surrounded by fibrosarcoma characterized by infiltrative growth (Fig. 5A), resembling phyllodes tumor of the breast (Fig. 5B). In some areas, the 2 tumor components are separated. These components were intermixed throughout the tumor, with tubular and spindle cells being the most predominant. The sarcoma cells were spindles of the same size with frequent mitoses (> 5/HPF) and arrayed as fascicular and interlaced (Fig. 5C). The nucleus was uniform and had little eosinophilic nuclei, and pathological karyokinesis was observed. Necrosis and ulcers can also be observed. Tumor metastasis tissues were histologically revealed in 5 of 15 lymph nodes, including adenocarcinoma and fibrosarcoma. The case had tumor of stage III with TNM clinical classification of T3N1M0.

**Figure 6:**
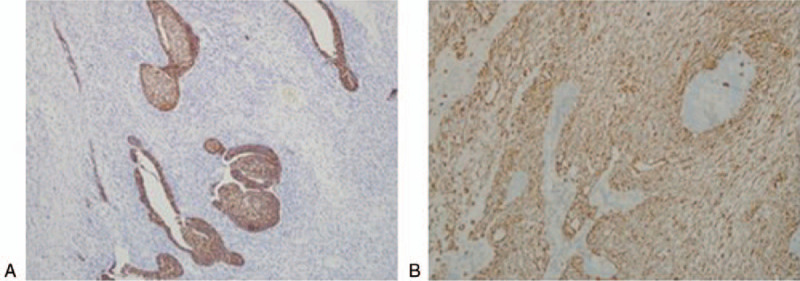
Immunohistochemical findings (Magnification: 20 × 10). A: The adenocarcinoma component was positive for pancytokeratin AE1/AE3 and CK8/18 and negative for vimentin. B: The spindle tumor component showed diffusely strong positive for vimentin.

Immunohistochemically, the adenocarcinoma component was positive for pancytokeratin AE1/AE3 and CK8/18 and negative for vimentin (Fig. 6A). However, the spindle tumor component was diffusely strongly positive for vimentin and negative for AE1/AE3 (Fig. 6B), whereas all the tumor cells were negative for neuroendocrine markers and other mesenchymal markers, such as desmin, SMA, myglobin, mygenin, S-100, DOG-1, CD34, CD117 and. Based on pathological examination, the final diagnosis was carcinosarcoma.

The patient was discharged on the 15th postoperative day in good condition. The patient died of cachexia and asphyxia caused by gastroesophageal reflux on July 8, 2013, 1 month after the operation.

### 2.3 Ethic statement

The study was approved by the ethics committee of the Second Hospital of Lanzhou University. Written informed consent was obtained from all the patients involved in the study.

## 3 Discussion

Carcinosarcoma and sarcomatoid carcinoma in the stomach are rare malignant tumors with high mortality, and 50% of patients die within 6 months after surgery.^[7]^ Carcinosarcoma is a biphasic tumor that simultaneously comprises adenocarcinoma (originating from epithelial tissue) and sarcoma (originating from mesenchymal/connective tissue) in a single tumor. Sarcomatoid carcinoma is a poorly differentiated carcinoma; in the upper gastrointestinal system, carcinosarcoma or sarcomatoid carcinoma is rarely reported, except in the esophagus.^[7]^

Sirsat et al defined a malignant tumor comprising both epithelial and mesenchymal elements as carcinosarcoma,^[8]^ which often occurs in various organs, such as salivary glands, lungs, uterus, pancreas, and esophagus, but rarely in the stomach. In 1904, the first case of gastric carcinosarcoma was reported by Queckenstadt.^[9]^ Until 2019, at least 69 cases of gastric carcinosarcoma have been published worldwide, mostly in Japan. The median age of the patients was 62 years (range, 29–80 years). The primary symptoms included epigastric pain, fatigue, swallowing difficulty, dysphagia, and weight loss. Moreover, most patients underwent surgical resection, and some underwent chemotherapy.

The appearance of gastric carcinosarcoma is divided into polypoid, exophytic, or endophytic, accompanied by ulcerative surfaces.^[10]^ Macroscopically, there were no significant differences between carcinosarcomas and gastric adenocarcinomas. Moreover, it is impossible to make an exact diagnosis based on the endoscope, CT, or MR.^[11]^

It can be diagnosed according to pathology, including morphology, by optical and immunohistochemistry. Microscopically, there are 2 types of carcinosarcoma reported:

1. true carcinosarcoma and
2. false carcinosarcoma or sarcomatoid carcinoma.^[12]^

The former is a mixed tumor comprising adenocarcinoma and true sarcoma, and the 2 components are closely related. As if they have a kind of causal relationship, sometimes, an obvious boundary between 2 neoplastic components can be seen, or 2 components are mixed together. They can be seen closely together or separated when transferring to lymphonodus of the stomach, just as the second case above-mentioned, Sarcoma cells are always negative for pancytokeratin AE1/AE3, but positive for mesenchymal marker vimentin, differentiating to cartilage, smooth muscle, bone, rhabdomyo and et al.^[9]^ Yoshida et al reported a 59-year-old man with extremely rare gastric carcinosarcoma with an osteoblastic component, and the patient died after 7 months. During autopsy, widespread metastases were present in the lungs, liver, lymph nodes, and peritoneum.^[13]^ Another gastric carcinomasarcoma with rhabdomyosarcomatous and neuroendocrine differentiation has also been reported.^[13–15]^ For example, Tsuneyama et al reported an unusual gastric carcinosarcoma with rhabdomyosarcomatous and neuroendocrinal differentiation in a 63-year-old Japanese male. They found that there were transitions between the sarcomatous and carcinoma elements. In addition, carcinoma cells with a cord-like or trabecular arrangement similar to that seen in endocrine carcinoma expressed chromogranin A.^[13]^ An 84-year-old man was diagnosed with carcinosarcoma in the esophagus and stomach. Tumors of the esophagus consist of spindle cell sarcoma and squamous cell carcinoma, and gastric tumors reveal spindle cell carcinoma. The patient died 7 months after the operation. Kuroda et al reported a 78-year-old man with gastric carcinosarcoma that was confined to the mucous membrane and positive for alpha-smooth muscle actin, calponin, and h-caldesmon. The patient had no recurrence for 2 years.^[9,16]^ Avivi et al reported the case of a 71-year-old man with stomach carcinosarcoma. The authors concluded that gastric carcinosarcoma should be considered as a part of the differential diagnosis when a large mass is found in the stomach. Histological identification is critical for appropriate surgical management, which is consistent with results of the present study.^[17,18]^

In contrast, sarcomatoid carcinoma is a malignant tumor that consists of adenocarcinoma and sarcomatous portions that stem from the carcinoma with evident transitional regions, just as in the first case. Both carcinoma and sarcomatoid spindle cell elements are positive for pancytokeratin AE1/AE3, while spindle cells are positive for vimentin.^[19]^ Occasionally, spindle cell carcinoma of the stomach presents with complicated symptoms, such as hematochezia and weight loss due to fistulous tract formation in the colon.^[20]^ In addition, a 76-year-old man had a 6 cm sarcomatoid carcinoma of the remnant stomach after distal subtotal gastrectomy with a duodenal ulcer 28 years ago.^[21]^

It is worth mentioning that both carcinosarcomas, sarcomatoid carcinomas, and spindle cell carcinomas are different from other biphasic tumors, such as some collision tumor, gastroblastoma, synovial sarcoma, and teratoma.^[8,22,23]^ A carcinosarcoma case was reported with C-kit expression^[19]^ and its sarcoma cells were partly and weakly positive for CD117, but no C-kit mutation was detected in exons 9, 11, 13, and 17, so the evidence was not sufficient to diagnose gastric carcinosarcoma with GIST, but carcinosarcoma with C-kit expression. GISTs originate from mesenchymal cells that control gut motility and can be classified as low or high risk according to several standards; therefore, we believe that gastric carcinoma combined with GIST should be classified as a collision tumor but not a true carcinosarcoma. In addition, gastroblastoma is a novel unique type of epitheliomesenchymal biphasic stomach tumor that was first reported in a series of 3 cases by Miettinen et al in 2009.^[24]^ It often arises in the stomachs of young people. The tumor consists of naïve but not sufficient atypia epithelial and mesenchymal components, the epithelial component is positive for cytokeratin, and the mesenchymal component is positive for vimentin and CD10. Subsequently, Shin et al described a case of gastric antrum in a 9-year-old boy in 2010.^[23]^ The tumor cells were not atypia enough to diagnose carcinosarcoma or sarcomatoid carcinoma.

In terms of morphology and immunohistochemistry, it is impossible to differentiate carcinosarcoma from sarcomatoid cancer of the stomach from primary gastric synovial sarcoma, which is also a rare, malignant, and biphasic tumor. It often occurs in young people and has been reported in more than 20 cases at present. Presently, the amplification of chimera transcription of YT-SSX1 or YT-SSX2 using FISH or RT-PCR is the most accurate method to diagnose primary gastric synovial sarcoma.^[24]^

Gastric carcinosarcoma is less metastatic, and the mortality rate is high.^[17]^ The 2 cases presented here revealed lymph node metastases. The second patient died just 1 month after surgery; however, our first case is still alive up to 2 and a half years after surgery by phone follow-up.

## 4 Conclusion

The present study provides insight into the clinical findings, diagnosis, and prognosis of carcinosarcomas and sarcomatoid carcinomas of the stomach. More cases are needed for further studies in the future.

## Author contributions

Li YP, Cui L, Chen Y, and Wang FR acquired data; Li YP, Cui L, and Wang FR analyzed data and drafted the article; Li YP and Wang FR made final approval of the article.

**Conceptualization:** Youpeng Li, Furong Wang.

**Data curation:** Youpeng Li, Li Cui, Ying Chen.

**Formal analysis:** Furong Wang.

**Funding acquisition:** Furong Wang.

**Investigation:** Youpeng Li, Ying Chen, Furong Wang.

**Methodology:** Li Cui.

**Supervision:** Furong Wang.

**Validation:** Ying Chen.

**Writing – original draft:** Youpeng Li, Li Cui.

**Writing – review & editing:** Furong Wang.

## References

[1] McCluggage WG. Malignant biphasic uterine tumours: carcinosarcomas or metaplastic carcinomas? J Clin Pathol 2002;55:321–5. 11986333 10.1136/jcp.55.5.321 PMC1769650

[2] Toyokawa G Takenoyama M Taguchi K. The first case of lung carcinosarcoma harboring in-frame deletions at exon19 in the EGFR gene. Lung Cancer 2013;81:491–4. 23891513 10.1016/j.lungcan.2013.06.013

[3] Berton-Rigaud D Devouassoux-Shisheboran M Ledermann JA. Gynecologic Cancer InterGroup (GCIG) consensus review for uterine and ovarian carcinosarcoma. Int J Gynecol Cancer 2014;24:S55–60. 25341582 10.1097/IGC.0000000000000228

[4] Vekony H Leemans CR Ylstra B. Salivary gland carcinosarcoma: oligonucleotide array CGH reveals similar genomic profiles in epithelial and mesenchymal components. Oral Oncol 2009;45:259–65. 18693132 10.1016/j.oraloncology.2008.05.009

[5] Darvishian F Sullivan J Teichberg S. Carcinosarcoma of the pancreas: a case report and review of the literature. Arch Pathol Lab Med 2002;126:1114–7. 12204065 10.5858/2002-126-1114-COTP

[6] Solerio D Ruffini E Camandona M. Carcinosarcoma of the esophagogastric junction. Tumori 2008;94:416–8. 18705412 10.1177/030089160809400320

[7] Sato Y Shimozono T Kawano S. Gastric carcinosarcoma, coexistence of adenosquamous carcinoma and rhabdomyosarcoma: a case report. Histopathology 2001;39:543–4. 11737318 10.1046/j.1365-2559.2001.1301e.x

[8] Sirsat MV Sampat MB. Collision tumour of the stomach. Indian J Cancer 1967;4:88–90. 6075537

[9] Yoshida H Tanaka N Tochigi N. Rapidly deforming gastric carcinosarcoma with osteoblastic component: an autopsy case report. World J Gastroenterol 2012;18:4064–8. 22912559 10.3748/wjg.v18.i30.4064 PMC3420005

[10] Kikuyama R Tanaka K Tano S. A case of gastric carcinosarcoma. Endoscopy 2009;41: (Suppl 2): E220–1. 19757363 10.1055/s-0029-1214937

[11] Choi KW Lee WY Hong SW. Carcinosarcoma of the stomach: a case report. J Gastric Cancer 2013;13:69–72. 23610722 10.5230/jgc.2013.13.1.69 PMC3627810

[12] Bekki T Fujikuni N Tanabe K. The gastric carcinosarcoma with severe venous invasion: a case report. Surg Case Rep 2018;4:14. 29380091 10.1186/s40792-018-0421-8 PMC5789122

[13] Tsuneyama K Sasaki M Sabit A. A case report of gastric carcinosarcoma with rhabdomyosarcomatous and neuroendocrinal differentiation. Pathol Res Pract 1999;195:93–7. discussion 98. 10093828 10.1016/S0344-0338(99)80077-6

[14] Melato M Bucconi S Grillo BP. Carcinosarcoma and separate neuroendocrine malignant tumor of a malignancy promoter, the gastric stump. Anticancer Res 1993;13:2485–8. 8135487

[15] Gohongi T Iida H Gunji N. Postsurgical radiation therapy for gastric carcinosarcoma with c-kit expression: a case report. World J Gastroenterol 2015;21:2830–5. 25759557 10.3748/wjg.v21.i9.2830 PMC4351239

[16] Kuroda H Saito H Kono Y. Carcinosarcoma of stomach confined to the mucosa. Yonago Acta Med 2017;60:246–50. 29434495 10.24563/yam.2017.12.006 PMC5803162

[17] Avivi E Yovel DZ Shirin H. Carcinosarcoma of the stomach. J Gastrointest Surg 2020;DOI: 10. 1007/s11605-020-04668-6:. 10.1007/s11605-020-04668-6 32519247

[18] Fujiie M Yamamoto M Taguchi K. Gastric carcinosarcoma with rhabdomyosarcomatous differentiation: a case report and review. Surg Case Rep 2016;2:52. 27250580 10.1186/s40792-016-0176-z PMC4889528

[19] An JW Cheung DY Seo MW. A case of spindle cell carcinoma of the stomach presenting with hematochezia and weight loss due to fistulous tract formation with colon. Korean J Gastroenterol 2013;62:126–30. 23981948 10.4166/kjg.2013.62.2.126

[20] Sato A Oki E Kohso H. Sarcomatoid carcinoma of the remnant stomach: report of a case. Surg Today 2013;43:308–12. 23238883 10.1007/s00595-012-0402-7

[21] Czerniak A Lotan G Engelberg IS. The simultaneous coexistence of adenocarcinoma and primary malignant lymphoma in the stomach. J Surg Oncol 1985;30:42–5. 4079417 10.1002/jso.2930300112

[22] Tokunaga O Morimatsu M Nakashima T. Collision tumor of the stomach with carcinosarcoma and tubulo-papillary adenocarcinoma. Acta Pathol Jpn 1979;29:819–24. 495098 10.1111/j.1440-1827.1979.tb00947.x

[23] Shin DH Lee JH Kang HJ. Novel epitheliomesenchymal biphasic stomach tumour (gastroblastoma) in a 9-year-old: morphological, ultrastructural and immunohistochemical findings. J Clin Pathol 2010;63:270–4. 20203230 10.1136/jcp.2009.074104 PMC2922722

[24] Kamata K Wada R Yajima N. Primary gastric synovial sarcoma: molecular diagnosis and prediction of prognosis. Clin J Gastroenterol 2013;6:303–8. 24707321 10.1007/s12328-013-0403-0 PMC3971440

